# The Anti-Inflammatory Role of Omega-3 Polyunsaturated Fatty Acids Metabolites in Pre-Clinical Models of Psychiatric, Neurodegenerative, and Neurological Disorders

**DOI:** 10.3389/fpsyt.2020.00122

**Published:** 2020-02-28

**Authors:** Juliette Giacobbe, Bonnie Benoiton, Patricia Zunszain, Carmine M. Pariante, Alessandra Borsini

**Affiliations:** ^1^Stress, Psychiatry and Immunology Laboratory, Department of Psychological Medicine, Institute of Psychiatry, Psychology & Neuroscience, King's College London, London, United Kingdom; ^2^Guy's King's and St. Thomas' School of Life Science and Medicine, King's College London, London, United Kingdom

**Keywords:** resolvin, protectin, maresin, neuroinflammation, omega-3, polyunsaturated fatty acid

## Abstract

Inflammation has been identified as one of the main pathophysiological mechanisms underlying neuropsychiatric and neurodegenerative disorders. Despite the role of inflammation in those conditions, there is still a lack of effective anti-inflammatory therapeutic strategies. Omega-3 polyunsaturated fatty acids (n-3 PUFAs) can reduce depressive symptoms and exert anti-inflammatory action putatively by the production of distinct n-3 PUFA-derived metabolites, such as resolvins D (RvD) and E (RvE) series, maresins (MaR) and protectins (PD), which are collectively named specialized pro-resolving mediators (SPMs) and act as strong anti-inflammatory agents. In this review we summarize evidence showing the effects of treatment with those metabolites in pre-clinical models of psychiatric, neurodegenerative and neurological disorders. A total of 25 pre-clinical studies were identified using the PubMed database. Overall, RvD and RvE treatment improved depressive-like behaviors, whereas protectins and maresins ameliorated neurological function. On a cellular level, RvDs increased serotonin levels in a model of depression, and decreased gliosis in neurodegenerative disorders. Protectins prevented neurite and dendrite retraction and apoptosis in models of neurodegeneration, while maresins reduced cell death across all studies. In terms of mechanisms, all SPMs down-regulated pro-inflammatory cytokines. Resolvins activated mTOR and MAP/ERK signaling in models of depression, while resolvins and maresins activated the NF-κB pathway in models of neurodegeneration and neurological disorders. Our review indicates a potential promising approach for tailored therapy with n-3 PUFAs-derived metabolites in the treatment of psychiatric, neurodegenerative, and neurological conditions.

## Introduction

Over the last few decades, inflammation has been identified as one of the main pathophysiological mechanisms underlying psychiatric conditions (1, 2). Indeed, over-expression of distinct pro-inflammatory cytokines, including interleukin 1 beta (IL-1β), IL-6, and tumor necrosis factor alpha (TNF-α), has been associated with several neuropsychiatric disorders, such as depression (3, 4), as well as neurodegenerative diseases, like Alzheimer's (AD) and Parkinson's (PD) (5, 6). In particular, patients with major depressive disorder (MDD) exhibit both increased immune activation and aberrant regulation of brain plasticity (7), which has been linked with abnormal cellular immunity (8). Similar abnormalities have also been reported in PD and AD, which are characterized by a dysregulated immune response, due to hyper-stimulation of microglia to activate distinct inflammatory signaling pathways (9) related to aggregates of alpha-synuclein and beta-amyloid protein, respectively (10, 11). In all these conditions, the presence of pro-inflammatory cytokines leads to the impairment of microglial function, including phagocytosis of debris, and propagation of inflammation (12). This is accompanied by an insufficient compensatory and regulatory function of anti-inflammatory cytokines, including IL-4, IL-10, and IL-13, which are produced by alternatively activated M2 microglia (13). Conversely, classically activated M1 microglia have been shown to be increased in the brain of patients (13, 14). Despite the role of inflammation in the context of both psychiatric and neurodegenerative disorders (15, 16), there is still a lack of effective anti-inflammatory strategies that are safe for everyday use and display a clear mechanism of action.

**Figure 1:**
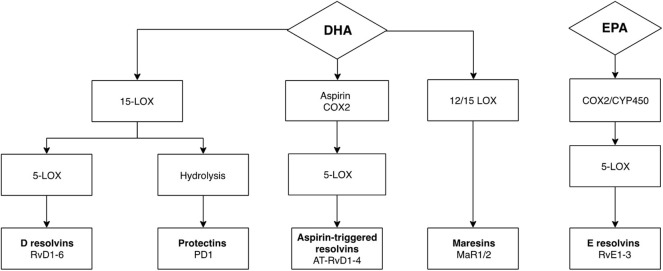
Metabolism of DHA and EPA to SPMs through enzymatic transformation. SPMs are produced upon metabolism of n-3 PUFAs by specific lipoxygenase and cyclooxygenase enzymes. Respectively, the enzymes 15-lipoxygenase-1 (15-LOX) and 12-lipoxygenase (12-LOX) are responsible for initiating the conversion of DHA to protectin-1 (PD1), and maresin 1 and 2 (MaR1, MaR2), whereas 15-LOX, cyclooxygenase 2 (COX-2) and cytochrome P450 are responsible for the conversion of DHA to resolvins D series (RvD), and of EPA to resolvins E series (RvE). Downstream, metabolism of RvD and RvE are dependent on 5-lipoxygenase (5-LOX). Aspirin-acetylated COX-2 followed by 5-lipoxygenase (5-LOX) transformation generates aspirin-triggered isomers of RvDs (AT-RvD).

Recently, increasing attention has been given to potentially anti-inflammatory nutritional interventions, particularly omega-3 polyunsaturated fatty acids (n-3 PUFAs), like eicosapentaenoic acid (EPA) and docosahexaenoic acid (DHA), which have been known to reduce depressive symptoms in patients (17, 18) and animal models (19), as well as cognitive symptoms (20). EPA has been found to be present at lower levels in patients with interferon-alpha-induced depression (21), the development of which has been shown to be prevented by EPA treatment (22), supporting n-3 PUFAs anti-inflammatory properties (23). Although the exact mechanisms underlying their mode of action remain unknown, n-3 PUFAs are important in regulating immune responses by inhibiting activation of pro-inflammatory pathways and reducing cytokine expression (24). This function has been suggested to be mediated by the production of distinct n-3 PUFAs-derived metabolites, defined as specialized pro-resolving mediators (SPMs), including resolvins D (RvD) and E (RvE) series, maresins (MaR) and protectins (PD), which become elevated upon exposure to an inflammatory challenge in order to re-establish internal immune homeostasis (25). In particular, SPMs are produced upon metabolism of n-3 PUFAs by specific enzymes including lipoxygenases, 5-lipoxygenase-1 (5-LOX), 12-LOX, and 15-LOX, cyclooxygenases, primarily COX-2, and cytochrome P450 enzymes (Figure 1). These enzymatic transformations occur rapidly within the organism and genetic variants of the involved enzymes have been associated with increased risk of developing interferon-alpha-induced depression (26), which suggests that the anti-inflammatory effects of n-3 PUFAs may indeed stem from SPMs actions.

Research into the effectiveness of SPMs treatment has been carried out in various models of peripheral and central inflammation. For example, RvDs and protectins have been shown to improve inflammatory outcomes in animal models of colitis and obesity-induced diabetes, where a reduction in cytokine levels, including IL-6, were reported in macrophages derived from bone marrow tissue (27, 28), as well as in adipose tissue (29). With respect to the CNS, evidence has shown that protectins and resolvins are produced in the brain, as shown by studies using brain tissue homogenates (30, 31), neuron-glia cultures or hippocampal tissue (32). In a model of inflammatory pain, RvDs and RvEs were found to reduce pain behaviors through central actions (33). Additionally, maresins have been demonstrated to attenuate mechanical allodynia (34, 35), a process involving central sensitization, and decreased levels of IL-1β, IL-6, and TNF-α in spinal cord tissue in models of neuropathic pain. Taken together, these findings therefore suggest the potential involvement of SPMs in other disorders within the CNS.

Given the need to elucidate the mechanisms whereby n-3 PUFAs-derived metabolites exert their anti-inflammatory actions and the potential role of SPMs in reducing CNS inflammation, it appears relevant to summarize the evidence provided thus far on their effects in the context of psychiatric, neurodegenerative, and neurological disorders, in addition to uncovering mechanisms specific to these conditions. Overall, 25 articles were obtained from the PubMed database, including *ex vivo, in vivo*, and *in vitro* studies investigating resolvins (RvD1, RvD2, RvE1, RvE2, RvE3), protectins (PD1, NPD1), and maresins (MaR1, MaR2) in relation to psychiatric, neurodegenerative, and neurological disorders affecting cognition, and in which neuroinflammation is part of the pathophysiology. Studies excluded from the search were or contained one or more of the following: not published in English language, did not look at the specific effects of treatment with resolvin, proctectin, or maresin, were not measuring psychiatric, neurological, neuroinflammatory, or cognitive outcomes.

## Behavioral, Cellular and Molecular Outcomes Identified Upon Treatment With SPMs

In this section of the review we summarize behavioral, cellular, and molecular outcomes identified in *ex vivo, in vivo*, and *in vitro* studies which used treatment with resolvins, protectins and maresins in the context of psychiatric, neurodegenerative, and neurological disorders (Table 1).

**Table 1:** Behavioral, cellular and molecular outcomes identified upon treatment with SPMs.

**Treatment**	**Type of study**	**Pathology**	**Model**	**Main findings**			**References**
				**Behavioral**	**Cellular**	**Mechanism**	
**Resolvin D**							
**RvD1**							
**Models of depression**							
RvD1	*In vivo*	Depression	LPS-induced, mice. I.c.v. RvD1 treatment and pathway antagonists	↘ TST immobility		Effects dependent on ALX/FPR2 rec., mTORC, MAP/ERK, AMPAR, PI3K/Akt	Deyama et al. (36)^*^
RvD1	*In vivo*	Myocardial infarct-associated depression	Rats I.c.v. RvD1 before or after ischemia	↗ social interaction ↘FST			Gilbert et al. (37)
RvD1 and AT-RvD1	*In vivo*	Fibromyalgia-associated depression	Resperine induced, mice. I.v. or i.c. RvD1 or AT-RvD1 treatment	AT-RvD1: ↘ mechanical allodynia (acute), nociception (chronic) ↘ FST immobility (chronic) RvD1: NS effect	AT-RvD1: ↗ dopamine (cortex), serotonin (thalamus), glutamate RvD1: NS effect		Klein et al. (38)^*^
RvD1	*In vivo*	Depression	Chronic unpredictable stress, mice. I.c.v. RvD1	↘FST, TST immobility			Ishikawa et al. (39)^*^
**Models of neurocognitive and neurological disorders**							
RvD1	*Ex vivo*	AD/MCI	PBMC of patients taking DHA+EPA supplements RvD1 treatment		RvD1 ↘ M1/M2 ratio in ApoE ε3/ε3 cells but ↗ M1/M2 ratio in ApoE ε3/ε4 cells		Famenini et al. (40)
RvD1	*Ex vivo*	AD	Macrophages from PBMC of patients taking DHA+EPA supplements Cells: DHA+EPA or RvD1 treatment Aβ incubation		RvD1 treatment: NS ↗ of phagocytosis compared with placebo	RvD1 treatment: NS ↗ of p-PERK expression NS ↘ caspase 3 expression in MCI patients	Olivera-Perez et al. (41)
RvD1	*Ex vivo*	AD	PBMC of AD patients. RvD1 treatment Aβ incubation Pre-treated with GPR32, EGTA, MEK1/2, PI3, or PKI antagonists		↗ phagocytosis ↘ caspase-3 dependent apoptosis	↗ phagocytosis dependent on GPR32, EGTA, MEK1/2, PI3, and PKI ↘ cytokines and chemokine transcription ↘ IL-1β, IL-6, IL-10, GMCSF, and TNF-α secretion	Mizwicki et al. (42)
AT-RvD1	*In vivo*	Traumatic brain injury	Midline perfusion injury, mice. I.p. AT-RvD1	↗ sensorimotor functions ↗ NOR task			Harrison et al. (43)^*^
AT-RvD1	*In vivo*	Surgery-induced cognitive decline	Open stabilized tibia fracture model, mice. Fear conditioning pre-surgery I.p. pre-treatment or delayed AT-RvD1	↗ memory ↗ impaired freezing behavior	Prevention of astrogliosis and prevention of ramification and ↘ cell area	↘ IL-6, LXA4 in plasma	Terrando et al. (44)
			Hippocampal slices from mice, post-surgery, and post-AT-RvD1 treatment		Pre-treatment ↗ short-term plasticity and LTP Delayed treatment ↗LTP		
RvD1	*In vitro*	Parkinson	Rat adrenal phaeochromocytoma cells, MPP+ -induced RvD1 treatment		↘ apoptosis, cellular damage ↗viability	↘ p-p38 MAPK, p-ERK ↘ NF-κB p50 ↘ TNF-α, not IL-6	Xu et al. (45)
RvD1	*In vitro*	AD	Human bone-marrow derived neuroblastoma cells RvD1 treatment		↘ apoptosis ↗viability	GPR32 expressed	Zhu et al. (46)^*^
			Embryonic human microglial cells RvD1 treatment Aβ42 incubation		↗ CD11b	GPR32 and ALX/FPR2 expressed	
**RvD2**							
**Models of depression**							
RvD2	*In vivo*	Fibromyalgia-associated depression	Resperine induced, mice. I.v. or i.c., acute or chronic.	↘ mechanical allodynia (acute), nociception (chronic) ↘ FST immobility (chronic)	↗ serotonin, glutamate		Klein et al. (38)^*^
RvD2	*In vivo*	Depression	Chronic unpredictable stress, mice. I.c.v. RvD2	↘ FST, TST immobility			Ishikawa et al. (39)^*^
RvD2	*In vivo*	Depression	LPS-induced, mice. I.c.v. RvD2 + pathway antagonists	↘ FST, TST immobility		Effects dependent on GPR18 rec., mTORC, MEK/ERK	Deyama et al. (36)^*^
**Models of neurocognitive and neurological disorders**							
RvD2	*In vivo*	Parkinson	LPS-induced, rats. I.c.v. RvD2	↗ motor behavior	↗ ramified microglia	↘ NF-κB ↘ IL-18, IL-6, NO, TNF-α, and IL-1β	Tian et al. (47)
	*Ex vivo*	Parkinson	Primary cortical microglia culture, rats LPS-induced RvD2 (5 ≠ concentrations)		↘ activated microglia	↘ NF-κB p65, iNOS, IkBa, IKKb ↘ IL-18, IL-6, NO, TNF-α, and IL-1β	
**Treatment**	**Type of study**	**Pathology**	**Model**	**Main findings**			**References**
				**Behavioral**	**Cellular**	**Mechanism**	
**Resolvin E**							
**Models of depression**							
RvE1	*In vivo*	Depression	LPS-induced, mice. I.c.v. RvE1, pathway antagonists	↘ FST, TST		Effects similar to ChemR23 agonist, dependent on mTORC1	Deyama et al. (48)
RvE2	*In vivo*	Depression	LPS-induced, mice. I.c.v. RvE2, pathway antagonists	↘ FST, TST		Effects similar to ChemR23 agonist	
RvE3	*In vivo*	Depression	LPS-induced, mice. I.c.v. RvE3	↘ TST			Deyama et al. (49)
**Models of neurocognitive and neurological disorders**							
RvE1	*In vivo*	Traumatic brain injury	Midline perfusion injury, mice. I.p. RvE1	↗ sleep	↗ ramified microglia, ↘ M1		Harrison et al. (43)^*^
RvE1	*In vivo*	AD	5xFAD mice. I.p. RvE1, LXA4, or RvE1+LXA4		RvE1+LXA4 ↘ microgliosis and astrogliosis RvE1+LXA4 ↘ Aβ40 RvE1 ↘ Aβ42 ↘ RvE1 and RvD2 in AD vs. WT, ↗ after RvE1 treatment	All ↘ GMCSF, IL-1β, IL-6, IL-10	Kantarci et al. (50)
**Treatment**	**Type of study**	**Pathology**	**Model**	**Main findings**			**References**
				**Behavioral**	**Cellular**	**Molecular/mechanism**	
**PROTECTIN**							
**Models of neurocognitive and neurological disorders**							
AT-PD1-SS AT-PD1-ME	*In vivo*	Ischemic stroke	Right middle cerebral artery occlusion, rats. I.v. AT-PD1-SS or AT-PD1-ME	↗ neurological recovery	↘ activated microglia/macrophages ↗ blood vessel + GFAP-rich scar density		Bazan et al. (51)
PD1	*In vivo*	TBI	Skull thinning in *fat-1* mice Normal or high n-6 diet I.v. DHA or i.c.v. PD1		↗ parenchymal cell survival in WT PD1 ↗ PD1 in fat-1 mice vs. WT		Ren et al. (52)
PD1	*In vivo*	Ischemic stroke	Right middle cerebral artery occlusion, rats. I.v. DHA, saline, PD1, or CSF treatment	↗ neurological score	↗ neuro- and angiogenesis ↘ IgG immunoreactivity ↗ axonal sprouting		Belayev et al. (53)
PD1 _n−3__DPA−ME_	*In vivo*	Epilepsy	Kainic acid epilepsy model, mice. I.c.v. PD1 after status epilepticus	Rescued ORT exploration time ↘ number of seizures	↘ astro- and microgliosis ↘ ectopic DCX cells No neuroprotection	↘ IL-1β, TNF-α mRNA	Frigerio et al. (54)
PD1	*In vitro*	PD	Primary rat dopaminergic mesencephalic neurons MPP+, MPTP, or rotenone induced PD1 treatment		↘ apoptosis in MPP+ and rotenone cells ↗ arborization (MPP+ cells only) ↘ dendrite retraction (MPP+, MPTP)		Calandria et al. (55)
PD1	*In vitro*	AD	Cortical human neuron-glia co-culture		↘ Aβ_42_-induced apoptosis ↘ neurite retraction	↗ Bcl-xl, Bcl-2, Blf(A1) ↘ Bax, Bik	Lukiw et al. (32)
PD1	*In vitro*	AD	Human neuronal-glial cells Challenged with Aβ42 oligomeric peptide or transfected with beta amyloid precursor protein (βAPP)_sw_ PD1 treatment		↘ Aβ_42_-induced apoptosis PD1 ↘ viability and ↗ apoptosis and cytotoxicity ↘ BACE1 ↗m-ADAM10 ↘ sAPPβSW ↗sAPPα	NPD1 mimics PPARγ receptor effects ↘ COX-2, TNF-α, B94 ↘ caspase-3	Zhao et al. (56)
PDX	*In vitro*	Ischemia	Mouse subventricular zone NSC Healthy or glucose-deprived PDX or DHA treatment		PDX ↘ proliferation in healthy NSC, ↗ proliferation in OGD NSC ↗ differentiation in healthy NSC (trend level) and OGD cells		Lo Van et al. (57)
PDX	*In vitro*	AD	Human bone-marrow derived neuroblastoma cells PDX treatment		↘ apoptosis ↗viability		Zhu et al. (46)^*^
**Treatment**	**Type of study**	**Pathology**	**Model**	**Main findings**			**References**
				**Behavioral**	**Cellular**	**Mechanism**	
**MARESIN**							
**Models of neurocognitive and neurological disorders**							
MaR1	*In vivo*	Stroke	MCAO, mice. I.c.v. MaR1 administration	↗ neurological score	↘ neurodegeneration, cell death (PSD95, synapsin1) ↘ gliosis	↘ NF-κB p65 ↘ TNF-α, IL-1β, MCP-1	Xian et al. (58)
MaR1	*In vitro*	ALS	SOD1 or TDP-43 expression in human neuroblastoma spinal cord cells H_2_O_2_ stress-induced cell death model DHA or MaR1 treatment		↘ cell death (MaR1 stronger than DHA) in SOD1/TDP-43 model ↘ oxidative stress-induced cell death	Caspase 3/7 inhibition by MaR1 ↘ ROS, ↘ p-NF-κB	Ohuchi et al. (59)
MaR1	*In vitro*	AD	Human bone-marrow derived neuroblastoma cells MaR1 treatment Embryonic human microglial cells Aβ42 incubation MaR1 treatment		↘ apoptosis ↗phagocytosis	↘ CD11b, MHC-II, CD86, CD40, and CD33	Zhu et al. (46)^*^

*15-LOX, 15-lipoxygenase; 5-LOX, 5-lipoxygenase; AD, Alzheimer's disease; ALX/FPR2, N-formyl peptide receptor 2; AMPAR, α-amino-3-hydroxy-5-methyl-4-isoxazolepropionic acid receptor; ApoE, Apolipoprotein E; AT-PD1-ME, aspirin- triggered protectin D1 methyl-ester; AT-PD1-SS, aspirin- triggered protectin D1 sodium-salt; AT-RvD1, aspirin-triggered resolvin D; Aβ, beta amyloid; BACE1, beta-secretase 1; CCL, CC chemokine ligand; ChemR23, chemokine-like receptor 1; CNS, central nervous system; COX-2, cyclooxygenase 2; CSF, cerebrospinal fluid; CXCL, chemokine C-X-C motif ligand; DCX, doublecortin; DHA, docosahexaenoic acid; EAE, experimental autoimmune encephalitis; EGTA, ethylene glycol tetraacetic acid; FST, forced swim test; GFAP, glial fibrillary acidic protein; GPR18, G protein-coupled receptor 18; GPR32, G protein-coupled receptor 32; GSH, glutathione; GSMCSF, granulocyte-macrophage colony-stimulating factor; Hcb, hemicerebellectomy; i.c., intrathecal; i.c.v., intracerebroventricular; IFN-γ, interferon gamma; IgG, immunoglobulin; IKK, IκB kinase; IL-1β, interleukin 1 beta; IL-6, interleukin 6; i.p., intraperitoneal; i.v., intravenous; LPS, lipopolysaccharide; LTP, long term potentiation; LXA4, lipoxin 4; MAPK, mitogen-activated protein kinase; MaR1, maresin 1; MCI, mild cognitive impairment; MCP-1, monocyte chemoattractant protein 1; MEK, mitogen-activated protein kinase; MHC-II, majoe histocompatibility complex class II; MPP+, 1-methyl-4-phenylpyridinium; MPTP, 1-methyl-4-phenyl-1,2,3,6-tetrahydropyridine; mTORC, mammalian target of rapamycin complex; n-3 PUFA, omega-3 polyunsaturated fatty acid; NF-κB, Nuclear factor-kappa B; NO, nitric oxide; NOR, novel object recognition task; NOS, nitric oxide synthases; NSC, neural stem cells; PBMC, peripheral blood mononuclear cells; PD1, protectin 1; PDX, protectin DX; p-ERK, phosphorylated extracellular signal–regulated kinase; PI3, phosphatidylinositide 3-kinase; PKI, protein kinase inhibitor; p-p38, phosphorylated p38; PPARγ, peroxisome proliferator-activated receptor gamma; p-PERK, phosphorylated protein kinase-like endoplasmic reticulum kinase; PSD95, postsynaptic density protein 95; RvD1, resolvin D 1; RvD2, resolvin D 2; RvE1, resolvin E 1; SCI, spinal cord injury; SOD-1, superoxide dismutase 1; SMNΔ7, survival motor neuron gene lacking exon 7; SPM, specialized pro-resolving mediators; TDP-43, TAR DNA-binding protein 43; TH+, tyrosine hydroxylase positive; TNF-α, tumor necrosis factor alpha; TST, tail suspension test; WT, wild type; βAPP_sw_, beta amyloid precursor protein swedish double mutation*.

↗ *increase*;

↘ *decrease*;

* *article appearing several times*.

### Resolvin D

#### RvD1

##### Behavioral findings

*Models of depression*. While depression has a wide range of symptoms, from persistent sad mood to appetite or sleep changes (60), it was assessed by behavioral despair, measured using the immobility time in the forced swim test (FST) or tail suspension test (TST) in most of the studies. In a mouse chronic unpredictable stress (CUS) model (39) intracranial RvD1 administration decreased behavioral despair in the FST. This was also found in a rat post-myocardial infarct model of depression, where depression-like behaviors are increased after occlusion of the left anterior descending coronary artery (37). However, neither peripheral nor central RvD1 administration improved FST immobility in a mouse fibromyalgia-induced depression model (38), where mice develop depression-like behavior after reserpine injection. In the TST, intracranial RvD1 also reduced behavioral despair in both CUS (39) and lipopolysaccharide (LPS)-induced mouse models of depression (36). Social behavior, commonly affected in depression, was enhanced by intracranial injection of RvD1 in a rat model of depression (37).

*Models of neurodegenerative and neurological disorders*. The behavioral outcomes of aspirin-triggered isomer of RvD1 (AT-RvD1) administration were investigated in two *in vivo* studies. Peripheral AT-RvD1 injection ameliorated sensorimotor function and memory after traumatic brain injury (TBI) in mice, confirming the hypothesis that reducing the prolonged inflammation caused by TBI would in consequence limit the impact seen in neurological functions (43). Peripheral AT-RvD1 administration was also beneficial on cognitive impairment and fear-associated freezing in mice with surgery-induced cognitive decline, mimicking the cognitive dysfunctions observed in some patients after orthopedic surgery (44).

##### Cellular findings

*Models of depression*. Only one of the studies previously mentioned investigated the cellular effects of AT-RvD1 in the context of depression. *In vivo*, intravenous AT-RvD1 administration increased levels of cortical dopamine and glutamate, and limited serotonin depletion in a mouse model of fibromyalgia-associated depression, suggesting a positive effect of treatment on neurotransmitter imbalance in depression (38).

*Models of neurodegenerative and neurological disorders*. Three studies investigated the effects of RvD1 in macrophages isolated from peripheral blood mononuclear cells (PBMC) of AD patients treated with n-3 PUFAs supplementation. In one study, RvD1 incubation of PBMC from AD patients improved phagocytosis of Aβ peptides on a trend level (41). In another, RvD1 significantly increased phagocytosis and decreased apoptosis in PBMC (42). In the third paper treatment with RvD1 decreased the M1/M2 macrophage ratio in PBMC from AD patients with the apolipoprotein E (APoE) ε3/ε3 genotype, while RvD1 increased it in cells with the APoE ε3/ε4 genotype (40).

*In vivo*, peripheral AT-RvD1 administration prevented astrogliosis and improved short and long-term potentiation in the hippocampus of mice with cognitive decline (44). *In vitro*, embryonic human microglia incubated with Aβ_42_ peptides and exposed to RvD1 had decreased expression of microglia pro-inflammatory markers CD11b and CD40 (46). In an *in vitro* model of PD using the toxin 1-methyl-4-phenyl pyridinium (MPP+) to target dopaminergic cells, RvD1 treatment of rat adrenal phaeochromocytoma cells rescued them from apoptosis (45).

##### Mechanisms of action

*Models of depression*. In the selected papers, only one *in vivo* study using a mouse model of depression examined the mechanisms underlying the actions of RvD1. The anti-depressant effects of intracranial administration of RvD1 were shown to be mediated by the activation of the N-formyl peptide receptor 2 (ALX/FPR2). Downstream, RvD1 was shown to act through activation of mammalian target of rapamycin complex 1(mTORC1), MAP/ERK, PI3K/Akt signaling, as well as by α-amino-3-hydroxy-5-methyl-4-isoxazolepropionic acid (AMPA) receptor (36).

*Models of neurodegenerative and neurological disorders*. Out of the five studies investigating the mechanisms of RvD1 in neurodegenerative and neurological disorders, two were *ex vivo*, one was *in vivo* and two were *in vitro*. In PBMC from AD patients, RvD1 treatment decreased the transcription of immune genes and the secretion of cytokines, such as IL-1β, IL-10, or IL-6 (42). In the same study, inhibition of the G protein-coupled receptor 32 (GRP32) prevented RvD1-induced phagocytosis of Aβ (42). In another study using PBMC from AD patients receiving oral nutritional intervention with n-3 PUFAs, cell treatment with RvD1 lowered p-PERK and caspase-3 expression on a trend level (41).

*In vivo*, IL-6 was decreased by peripheral RvD1 injection, along with the n-6 PUFAs-derived SPM lipoxin (LXA_4_) in the plasma of mice with surgery-induced cognitive decline (44). *In vitro*, RvD1 reduced TNF-α protein expression, but not IL-6, and prevented high levels of NF-κB p50 in a PD model of rat adrenal phaeochromocytoma cells (45). The expression of GRP32 was also confirmed in human bone-marrow derived neuroblastoma cells (46).

#### RvD2

##### Behavioral and cellular findings

*Models of depression*. In three mouse models of depression, RvD2 was shown to have positive effects on depressive-like behavior, however, only one study also investigated cellular outcomes. Central RvD2 administration was reported to improve FST and TST scores in LPS-induced (36) and in a CUS model of depression (39). Similarly, in a model of fibromyalgia-associated depression, intravenous RvD2 prevented immobility in the FST (38). With respect to cellular findings, RvD2 administration partially prevented total brain serotonin loss and increased glutamate levels (38).

*Models of neurodegenerative and neurological disorders*. To our knowledge, only one study described findings on the behavioral and cellular effects of RvD2 administration in neurodegenerative disorders. In a LPS-induced PD model, intracranial addition of RvD2 to apomorphine, a non-selective dopamine receptor agonist, improved motor function of rats more efficiently, when compared with apomorphine alone (47). Regarding cellular findings, RvD2 effectively reduced the number of activated microglia and increased the ramified phenotype in the substantia nigra of rats with PD. This was also shown in a primary culture of cortical microglia from neonatal rats (47).

##### Mechanisms of action

*Models of depression*. Among the studies previously mentioned, only one investigated the mechanisms underlying the effects of treatment with RvD2 in a model of depression. In particular, they showed that improvement in depressive-like behavior was observed in mice after intracranial RvD2 administration, which was independently mediated by GPR18, a G-protein-coupled receptor activated by cannabinoids (61) and RvD2, mTORC1, and MAP/ERK signaling (36).

*Models of neurodegenerative and neurological disorders*. In one study, RvD2 was reported to exert its beneficial actions through microglia in LPS-induced PD models. Specifically, RvD2 decreased transcription of several cytokines such as IL-18, IL-6, TNF-α, and IL-1β in the cytoplasm in an *in vitro* model of PD using rat primary cortical microglia. The expression of these cytokines was also reduced in the plasma of PD rats after central injection of RvD2. Moreover, RvD2 effectively prevented an up-regulation of NF-κB p65 subunit and IκBα in ventral mesencephalon microglia of PD rats (47).

The evidence summarized in this section highlights the role of RvDs in reducing depression-like behavior in models of depression, and in decreasing glial inflammatory processes in neurogenerative models.

### Resolvin E

#### Behavioral and Cellular Findings

##### Models of depression

RvE series were shown to have beneficial effects in mice when injected centrally. Administration of RvE1, RvE2, and RvE3 improved behavioral despair in the TST in a LPS-induced model of depression (48, 49). This was also demonstrated in the FST, but only in respects of intracranial RvE1 and RvE2 injection (49).

##### Models of neurodegenerative and neurological disorders

One *in vivo* study investigated the behavioral effects of RvE1, and two *in vivo* studies investigated the cellular effects. In a mouse model of TBI, peripheral RvE1 administration affected sleep during the first 12 h post-injury. Specifically, an overall increase in number, but not length, of sleep bouts in both light and dark periods was seen upon RvE1 administration (43). On a cellular level, RvE1 administration increased the number of ramified microglia and decreased the number of rod microglia in the primary somatosensory cortex of mice (43). In addition, intraperitoneal injection of RvE1 with LXA_4_ decreased microgliosis and astrogliosis in the cortex and hippocampus of AD mice (50).

#### Mechanisms of Action

##### Models of depression

One *in vivo* study proposed two different mechanisms of actions for RvEs using a model of LPS-induced depression in mice. Firstly, intracranial injection of RvE1 and RvE2 produced anti-depressant effects similar to those observed by activating ChemR23, a G-coupled receptor activated by chemerin (62) and RvE1 (63), suggesting the involvement of this receptor in depression. Secondly, inhibition of the mTORC1 pathway was able to prevent the anti-depressant effects of RvE1 (49).

##### Models of neurodegenerative and neurological disorders

In an *in vivo* transgenic mouse model of AD, RvE1 was shown to exert its effects through down-regulation of various pro-inflammatory factors. Specifically, peripheral RvE1 injection reduced levels of IL-6, IL-1β, IL-10, granulocyte-macrophage colony-stimulating factor (GM-CSF), IFN-γ, TNF-α, monocyte chemoattractant protein 1 (MCP-1), macrophage inflammatory protein (MIP)-1a, and MIP1b in the prefrontal cortex (50).

The evidence summarized in this section supports the potential of RvEs, similar to RvDs, to alleviate depression-like behavior, which would occur via mTORC1 activation. In terms of neurodegenerative disorders, studies clearly present RvEs as beneficial agents against the increased levels of cytokines and pro-inflammatory factors present in those conditions.

### Protectins

#### Behavioral Findings

##### Models of neurodegenerative and neurological disorders

Behavioral effects of PD1 administration were measured in three *in vivo* studies, one in the context of epilepsy and two in the context of stroke, both conditions which are associated with increased central inflammation affecting neurogenesis-related cognitive processes. Intracranial PD1 administration improved cognitive function, specifically non-spatial recognition memory, in the novel object recognition task in kainic acid-induced epilepsy in mice (54). PD1 also reduced frequency and seizure duration and prevented weight loss (54). Additionally, intravenous injection of PD1 and its aspirin-triggered isomer (AT-PD1) improved neurological recovery in rat models of ischemic stroke using middle cerebral artery occlusion (51, 53).

#### Cellular Findings

##### Models of neurodegenerative and neurological disorders

Cellular outcomes were investigated in nine studies both *in vivo* and *in vitro*, predominantly using models of AD and ischemia. Intravenous administration of PD1 *in vivo* reduced immunoglobulin G (IgG) immunoreactivity in the cortex, subcortex, and whole right hemisphere of rats subject to ischemic stroke (53). It also inhibited astrocyte and microglia activation in the penumbra of ischemic rats (51). Likewise, intracranial infusion of PD1 in epileptic mice decreased astrogliosis and microgliosis in the hippocampus, and increased neuroblasts migration in the hilus (54). In a mouse model of TBI, intracranial administration of PD1 also improved parenchymal cell survival (52).

*In vitro*, PD1 treatment decreased Aβ_42_ production (56) and prevented Aβ_42_-induced apoptosis and increased cell viability in two human models of AD, both using cortical neuron-glia co-culture (32, 56). This was also observed upon treatment with protectin isomer, PDX, in a human bone-marrow derived neuroblastoma cell model of AD (46). In a rat dopaminergic mesencephalon neurons model of PD, PD1 treatment decreased dendritic retraction and increased neuronal survival (55). Finally, in an *in vitro* model of ischemia, PDX also increased proliferation of mice subventricular zone neural progenitors (57).

#### Mechanisms of Action

##### Models of neurodegenerative and neurological disorders

One *in vivo* and two *in vitro* studies investigated the mechanisms of PD1. *In vivo*, transcription and expression of IL-1β and TNF-α were reduced in the hippocampus upon PD1 intracranial administration in a murine model of epilepsy (54). In an *in vitro* model of AD, PD1 administration reduced Aβ_42_ production through repression of pro-inflammatory molecules, including COX-2 and TNF-α (56). Furthermore, PD1 enhanced expression of anti-apoptotic proteins of the B-cell lymphoma 2 (Bcl-2) gene family (32) and reduced caspase-3 activity in cortical human neuronal cells *in vitro* (56).

Based on the evidence summarized in this section, protectins are especially useful in reducing behavioral deficits observed in neurological disorders, most likely via reducing microgliosis and pro-inflammatory cytokines levels.

### Maresins

#### Behavioral and Cellular Findings

##### Models of neurodegenerative and neurological disorders

One *in vivo* study investigated the behavioral effects of treatment with MaR1, whereas three *in vitro* studies assessed cellular outcomes. In an *in vivo* mouse model of stroke, intracranial administration of MaR1 reduced neurological impairments over time (58). On a cellular level, administration of MaR1 protected against brain cell death and inhibited the degradation of postsynaptic density protein 95 (PSD95) and synapsin. Furthermore, MaR1 administration also inhibited neutrophil infiltration and glial activation in the cortex (58). *In vitro*, MaR1 treatment prevented cell death in human bone-marrow derived neuroblastoma cell models of ALS and AD (46, 59). MaR1 also stimulated an increase of Aβ_42_ phagocytosis in embryonic human microglial cells (46).

#### Mechanisms of Action

##### Models of neurodegenerative and neurological disorders

All three studies previously mentioned investigated the mechanisms of action of PD1. In an *in vivo* mouse model of stroke, expression of TNF-α, IL-1β, and MCP-1 in the cortex was reduced by intracranial administration of MaR1. Furthermore, MaR1 decreased NF-κB activation through down-regulation of p65 phosphorylation (58). Similar effects were seen *in vitro*, with MaR1 treatment decreasing levels of phosphorylated NF-κB in human bone-marrow derived neuroblastoma cells (59). MaR1 treatment of embryonic human microglia also induced a reduction in pro-inflammatory markers including CD11b, major histocompatibility complex class II (MHC-II), CD86, CD40, and CD33 (46).

The limited evidence available on maresins suggests that they might benefit neurological conditions, specifically by reducing cell death and inflammatory factors, which may be related to decreased NF-κB pathway activation.

## Overall Discussion of the Evidence

**Figure 2:**
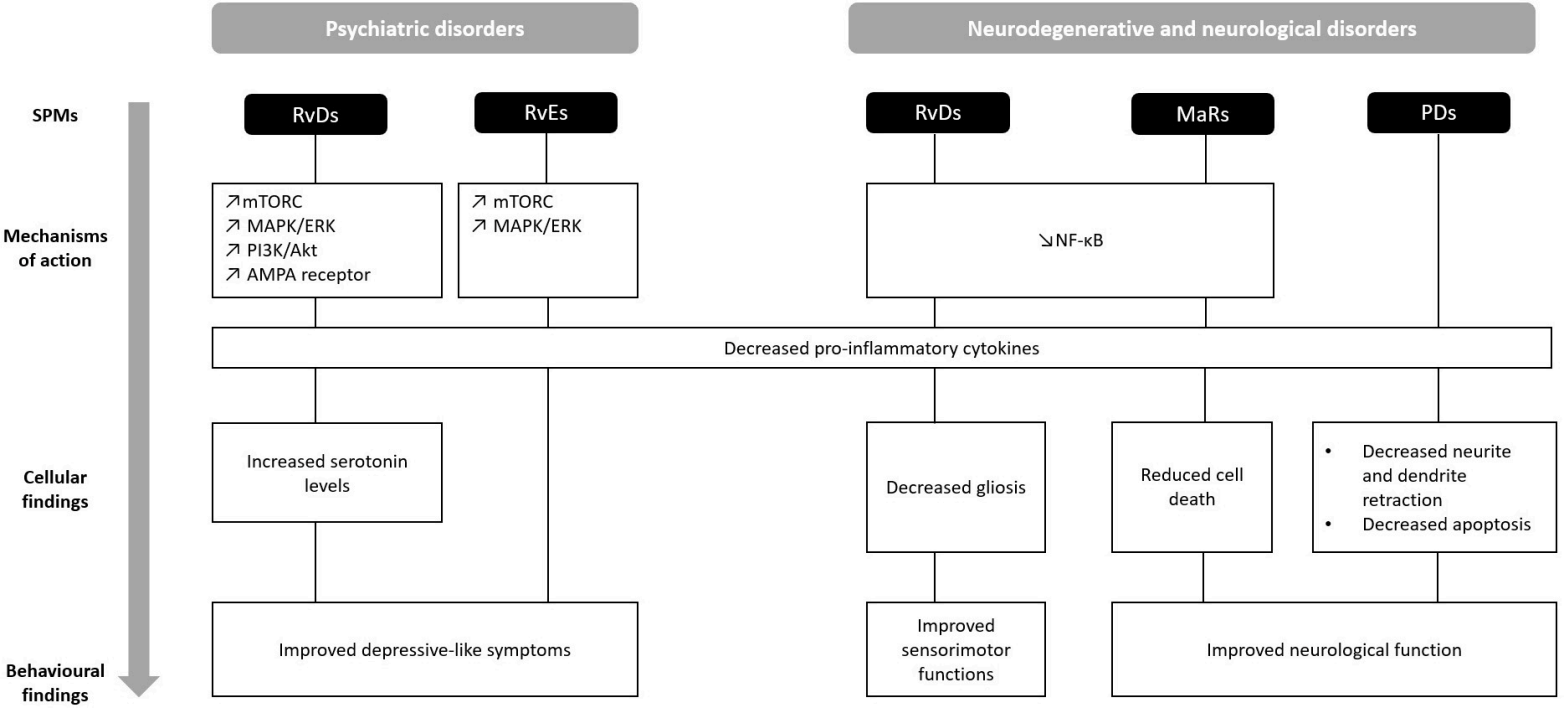
Comparison of behavioral, cellular, and molecular findings upon treatment with SPMs in the context of psychiatric, neurodegenerative, and neurological disorders. AMPA, α-amino-3-hydroxy-5-methyl-4-isoxazolepropionic acid; MAP/ERK, mitogen-activated protein kinases/extracellular signal-regulated kinases; MaR, maresin; mTORC, mammalian target of rapamycin complex; NF-κB, Nuclear factor-kappa B; PD, protectin; PI3K/Akt, Phosphoinositide 3-kinases/Protein kinase B; RvD, resolvins D series; RvE, resolvins E series. ↗ increase; ↘ decrease.

This review summarizes evidence on the beneficial effects of resolvins, protectins and maresins, in the treatment of psychiatric, neurodegenerative, and neurological disorders (Figure 2). Overall, treatment with both RvD and RvE improved depressive-like behaviors in various animal models of depression, whereas PD1 and MaR1 ameliorated neurological function. On a cellular level, RvD1 and RvD2 increased serotonin levels in a model of depression, and decreased gliosis in neurodegenerative disorders. In contrast, PD1 and PDX prevented neurite and dendrite retraction and apoptosis in models of neurodegeneration, while MaR1 reduced cell death across all studies. In terms of mechanisms, all SPMs down-regulated pro-inflammatory cytokines, such as IL-1β, IL-6, and TNF-α. RvD1, RvD2, and RvE1 exerted their effects through mTOR and MAP/ERK signaling in models of depression, while RvD1, RvD2, and MaR1 through the NF-κB pathway in models of neurodegeneration and neurological disorders. These findings suggest that not only do SPMs have anti-inflammatory properties across different models, but also possess characteristic therapeutic effects depending on the condition.

Despite the scarce number of studies conducted in psychiatric disorders, differences among specific SPMs could be drawn on several levels. In particular, RvD1 and RvEs were the most effective in improving depressive symptoms across several mouse models (36, 39, 48, 49). This could be explained by their mechanistic actions, which were notably distinct between psychiatric and neurological conditions. The mTORC1 pathway, which is a key signaling pathway in the effectiveness of antidepressants (64), was found to underlie the behavioral effects of resolvins (36, 49). Similar findings were presented for the MAPK/ERK pathway and PI3K/Akt and AMPA signaling (36), which are involved in cell growth and proliferation and can influence the expression of proteins associated with gene × environment interactions in depression (65). Moreover, all of these are key elements involved in neurogenesis (66), which is impaired by pro-inflammatory cytokines (67) and has been shown to be rescued by n-3 PUFAs treatment after IL-1β challenge *in vitro* (68).

With respect to neurodegenerative disorders, none of the SPMs could be distinguished in terms of better therapeutic effects. While apoptosis or gliosis were equally reduced by RvD1, RvE1, PD1, and MaR1 in *in vivo* and *in vitro* models, the benefits observed in *ex vivo* studies using patient-derived cells remained on a trend level (41) or were restricted to specific sub-groups (40). Although it is difficult to disentangle the underlying cause of these seemingly puzzling findings, the situation can be closely related to the reality of research into AD therapy. Many anti-inflammatory drugs appear promising at pre-clinical stages but are not effective in clinical trials, presumably due to the complexity of the disorder and the number of interacting factors (11). Further investigation is thus necessary to achieve a clearer understanding of SPMs in neurodegenerative disorders.

Although, maresins and protectins have not been examined in the context of depression, the evidence was conclusive in neurological disorders, where they appear to have a greater potential. PD1, PDX, and MaR1 improved neurological function in animal models of ischaemia, and TBI (51, 53, 58). In line with this, PD1 limited cell death, highlighting its neuroprotective abilities. MaR1 likely had a greater effect in these conditions due to its presence in macrophages and its more potent role in dampening the activation of microglia (69), which are more acutely and severely triggered in those conditions. Additionally, MaR1 promotes tissue regeneration, which could be of increased therapeutic value in ischemic stroke (35).

Thus, the ability of specific metabolites to improve behavioral, cellular and mechanistic components differentially in psychiatric and neurodegenerative disorders could be a basis for new personalized therapeutic strategies. Although current pharmacotherapies for AD and PD appear to slow the progression of cognitive impairment, the benefits have often found to be marginal and non-sustained (70). Additionally, up to one third of MDD patients fail to respond to first-line pharmacological treatment (71), which has been associated with elevated plasma pro-inflammatory factors expression (72). With AD projected to hit 131 million people by 2050 (73) and depression affecting about 5% of the world's population (74), new treatment avenues are needed more than ever. N-3 PUFAs have been approved as safe when administered in doses up to 3 g per day and minor side-effects are rare (75). Recent reviews and meta-analysis have reported a clinical efficacy of n-3 PUFAs treatment, which might be partly attributable to SPMs, in MDD and AD patients (76, 77). More interestingly, the majority of the animal studies so far only used males, which have recently been shown to have higher baseline levels of n-3 PUFAs metabolites than females in brain tissue (78). The single study using female mice reported positive effects of RvE1 on inflammatory factors, however, this does not allow for direct comparison between sexes (50). With women being at increased risk of developing MDD and AD (79, 80), further insight into this question is necessary as they might even more particularly benefit from this type of intervention.

Based on the findings from our review, personalized SPMs treatment could be a therapeutic possibility. RvD1, RvD2, or RvE1 could prove to be beneficial in psychiatric conditions, like depression, while MaR1 or PD1 would be optimally targeted toward neurological conditions. Although more studies are required to determine their exact influence and production in the brain, our review indicates a potential promising approach for tailored therapy with SPMs. With further research, this could lead to subsequent dietary recommendations and nutritional interventions in the treatment of psychiatric, neurodegenerative or neurological conditions, as n-3 PUFAs have been demonstrated to raise specific SPMs levels (81).

This review has few limitations that must be considered, such as the number of studies meeting the inclusion criteria and the prominence of cognitive and neurological compared with psychiatric studies. Additionally, dosage and route of administration between metabolites was also variable. Nonetheless, this is the first review to compare the effects of SPMs in the context of psychiatric, neurodegenerative and neurological disorders and sheds light on the differential mechanisms mediating their beneficial properties. Further research is needed to elucidate the exact mechanisms of action of these metabolites, as well as the extent of their anti-inflammatory properties, in order to discern which disorder they should optimally target.

## Author Contributions

All authors listed have made a substantial, direct and intellectual contribution to the work, and approved it for publication.

### Conflict of Interest

The authors declare that the research was conducted in the absence of any commercial or financial relationships that could be construed as a potential conflict of interest.
